# Rational Design of Nanostructured Porous and Advanced Getter Materials for Vacuum Insulation Panels

**DOI:** 10.3390/nano15070532

**Published:** 2025-03-31

**Authors:** Juan Wang, Zhibin Pei, Ningning Zhou

**Affiliations:** 1 School of Mechanical and Electrical Engineering, Hefei Technology College, Hefei 230012, China; wangjuan@htc.edu.cn; 2 Materials Department, Advanced Research Center, Hefei Hualing Co., Ltd., Hefei 230000, China; 3 Key Laboratory of Materials and Technologies for Advanced Batteries, School of Energy, Materials and Chemical Engineering, Hefei University, Hefei 230601, China

**Keywords:** vacuum insulation panels, getter materials, nanoporous design, gas adsorption, long-term reliability

## Abstract

Vacuum insulation panels (VIPs) have emerged as a cutting-edge strategy for achieving superior thermal insulation across a wide range of applications, including refrigerators, cold-chain transportation and building envelopes. The key factor for the exceptional performance of VIPs is maintaining an ultralow pressure environment within the panels, which is crucial for minimizing heat transfer. However, the presence of non-condensable gases can compromise the vacuum state, leading to a reduced insulation effectiveness during a panel’s service life. This review offers a comprehensive analysis of getter materials used in VIPs, focusing on their fundamental properties, types, integration techniques and performance characteristics, further emphasizing the challenges and potential directions for the development of getter materials. Overall, this review intends to provide novel insights into the development of getter materials for use in VIPs, offering essential viewpoints to aid future studies on this topic.

## 1. Introduction

**Figure 1:**
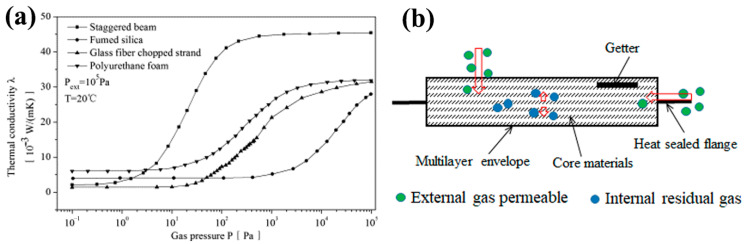
(**a**) Thermal conductivity of various core materials as a function of internal pressure. This figure was reproduced with permission from ref. [12]. (**b**) Schematic of a VIP structure and the vacuum leakage process. The red arrows indicate the direction of gas diffusion.

Refrigerators are one of the most prevalent household appliances, and their energy consumption makes up a significant amount of the overall household energy use, which is concerning given the growing concerns about global climate change and the growing emphasis on energy conservation. It is crucial to reduce energy consumption and mitigate environmental impact to improve the thermal insulation performance of refrigerators. Vacuum insulation panels (VIPs) with exceptional thermal resistance and compact sizes have emerged as a promising strategy to enhance the energy efficiency of refrigerators [1,2,3]. The long-term reliability of VIPs is essential for maintaining the energy efficiency and performance of refrigerators over time [4,5]. VIPs depend on the vacuum environment to minimize heat transfer; thus, any breach in the vacuum can lead to a significant decrease in their insulating properties, leading to increase heat transfer and reduce energy efficiency [6,7,8,9,10]. Figure 1a displays the thermal transport properties of VIPs across varying core materials and gas pressure regimes. The thermal conductivity curve reveals three distinct regimes governed by different heat transfer mechanisms. In the high-pressure regime (>10^4^ Pa), thermal conductivity remains pressure-independent due to the dominance of gas-phase conduction. A transitional regime (10^2^–10^4^ Pa) exhibits a steep conductivity reduction, primarily attributed to diminished gas convection effects. Below the critical threshold of 100 Pa, thermal conductivity stabilizes at remarkably low values (2–8 mW/(m·K)), where solid-phase conduction through the core matrix and radiative transfer become predominant. The inflection region between 5 and 100 Pa represents the operational pressure window for optimal VIP performance, as further vacuum enhancement (<100 Pa) provides diminishing returns in thermal resistance improvement [10,11,12,13,14]. Therefore, the retention of continuous vacuum integrity within the core structure is conserved to be a fundamental prerequisite for preserving the long-term thermal resistance properties of VIPs, especially in refrigeration applications involving decades of service reliability.

The VIP is characterized by a sandwich structure (Figure 1b). The main components consist of inner core materials, a multilayer envelope and getters. A deterioration in a panel’s long-term reliability performance is linked to the degradation of the vacuum degree, which can be attributed to the internal gases [4,15,16,17,18]. The gas sources primarily include residual air trapped during manufacturing, material outgassing (particularly from uncured components or volatile compounds accelerated by thermal aging), external gas permeation through micro-defects in barrier films and gas ingress via sealing imperfections (Figure 1b) [19,20]. Several strategies have been put forward to reduce the amount of gas in VIPs, including employing high-quality impermeable barrier materials [4,21,22,23,24,25,26], robust sealing techniques [27,28] and the inclusion of getters to absorb residual gases or outgassing from the panel materials [14,29,30,31]. While barrier films and sealing techniques address external gas ingress, internal gas generation from material outgassing and residual production artifacts necessitates complementary mitigation strategies. This challenge has catalyzed significant advances in functional getter materials capable of actively scavenging reactive gas species, forming the cornerstone of modern VIP longevity.

Advanced getter systems serve as an essential gas scavenger to uphold a high vacuum integrity in VIPs, significantly extending service lifetimes and ensuring ultra-low thermal conductivity [31,32,33]. At present, tremendous efforts have been devoted to improving the performance of getters, which can be categorized into three types: (1) Nanostructured getter materials are designed to provide many catalytic active sites for promoting the conversion kinetics, capturing gas molecules to the maximum extent [29,34,35,36]. (2) Alloyed materials are utilized to mitigate the diffusion of gas atoms on the material surface through chemical adsorption [11,30,37]; (3) the electrochemical method, a safer approach, has been introduced to enhance gas adsorption kinetics [38]. The above-mentioned methods are not used independently, usually requiring a combination of several methods. Even, it is still challenging to find highly active and cost-effective getter materials for mass production.

As we know, the escalating global demand for energy-efficient thermal management systems necessitates VIPs with multi-decadal service reliability. Nevertheless, comprehensive studies addressing the fundamental challenges have yet to be conclusively established in the literature. The fundamental challenges facing getter materials for prolonged VIP serviceability are as follows: (1) mechanistic understanding of gas sorption dynamics, (2) robust frameworks for lifetime performance prediction, and (3) systematic techno-economic assessments of novel getter materials. Our review comprehensively addresses these challenges through a synergistic integration of breakthroughs in gas-material interface physics, catalytic reaction pathways, and scalable synthesis strategies for advanced nanomaterials. This review commences with an examination of getter applications in VIPs, followed by a discussion of their prospective advantages in physicochemical mechanisms. We systematically evaluate recent advancements in getter material development for VIP applications, concluding with a critical assessment of current challenges and future research directions for next-generation getter systems. We believe that this review can provide useful insights for the rational design of a highly efficient getter material, further push forward the practical applications of VIPs.

## 2. Physical and Chemical Properties of Getter

The lifespan and performance of VIPs are intrinsically linked to the internal residual gas pressure and composition. The gas within VIPs originates from four pathways: (1) the substantial source is the residual air entrapped through the production process. Although many efforts have been made to evacuate the panel to a high vacuum, a residual quantity of air may persist, contributing to the initial gas load. (2) Undesired outgassing occurs from the component materials of VIPs over time, especially if the materials contain volatile compounds or are not fully cured. This outgassing can be exacerbated by temperature fluctuations and the materials aging. (3) Another gas source is permeable external gases. Despite being equipped with high-quality barrier films, it may exist tiny defects or pores enable air to permeate into sealed envelope materials to weaken the vacuum degree. (4) The sealing process can introduce gases into the VIP. If the seals are not entirely airtight or if there are defects in the sealing materials, air can leak into the panel, compromising the vacuum. These gas infiltration mechanisms collectively determine the thermodynamic environment within VIPs, where the gas composition critically influences thermal performance. Different gaseous species exhibit distinct thermophysical properties. There is a direct correlation between the types of residual gases (e.g., H_2_, H_2_O, CO_2_) and their partial pressures and vacuum degradation kinetics [14,39]. For instance, H_2_ has a thermal conductivity of 0.18 W/(m·K), which is approximately 7 times higher than that of air at 0.026 W/(m·K) [40]. Such disproportionate impacts stem from the quantum tunneling effect in H_2_ diffusion and its non-adiabatic interactions with core materials [41,42]. Thus, the accurate identification of the composition of residual gas within VIPs is an essential requirement to optimize designed materials and prolong the service life of panels, maintaining long-term thermal efficiency.

Quadrupole mass spectrometers are widely employed for residual gas analysis in high- and ultrahigh-vacuum environments, enabling a precise identification of gas species and the measurement of partial pressures ranging from 10^−4^ to 10^−11^ Pa [43,44]. Table 1 shows the main compositions of residual gases measured by quadrupole mass spectrometers. The residual gases within VIPs, particularly atmospheric constituents (e.g., N_2_, O_2_, H_2_O, and Ar), originate from three primary pathways: (1) gradual diffusion through the gas barrier envelope during service life, (2) retained gas molecules from incomplete vacuum extraction during manufacturing processes, and (3) the progressive release of adsorbed air layers from the core material’s porous surface structure [14,45]. H_2_ is likely derived from the breakdown of H_2_O due to permeation and diffusion processes within the gas barrier laminate, as well as from the degradation of organic substances [14,46]. Hydrocarbon species (C_x_H_y_) and compounds are predominantly sourced from the degradation of organics present in the gas barrier laminate and the core material, for example, the adhesive used to bond the gas barrier laminate or the sizing agent applied to the glass fiber surface [14,35]. The generation of CO and CO_2_ primarily stems from oxidation reactions between O_2_ and carbon-bearing components within VIPs, and the decomposition processes of organic materials under vacuum conditions [34,35,36,37,38,39,40,41,42,43,44,45,47]. This elevated H_2_ pressure may be mechanistically linked to the CO_2_ generation process, potentially through competitive adsorption–desorption equilibria or secondary reduction reactions involving carbon-containing species at material interfaces [48]. Although qualitative analysis provides preliminary insights into gas species identification, the quantitative analysis of residual gases within VIPs is still a significant technical challenge. Achieving accurate in situ detection and precise quantification of residual gas composition and concentration remains a critical direction for future research and technological advancement. Further development of advanced analytical methodologies is essential to address the complexities of gas phase interactions, aging mechanisms, and long-term performance evaluation in VIP systems.

The key to eliminating these residual gases is choosing the appropriate getter. The getters fitted in VIPs can effectively capture and fix the free residual gases through adsorption and reactions to maintain high-vacuum systems, thereby extending the lifespan of vacuum products [49]. The gas-capturing mechanisms of the getters can generally be classified into three categories. The first category is physical adsorbents characterized by substantial specific surface areas and hierarchically porous structures. These adsorbents exhibit selective adsorption capabilities toward gas molecules of differential kinetic diameters, which are primarily governed by their extensive surface interactions and microporous confinement effects. The adsorption process is facilitated by van der Waals interactions, allowing for efficient molecular trapping within their potential energy wells [50,51]. These forces are also evident in the interactions between atoms or molecules. Activated carbon and molecular sieves are prevalent materials utilized for physical adsorption [36,52,53]. The second type includes chemical getters that engage in regulated chemical reactions with adsorbed gaseous species. These systems primarily consist of transition metal oxides that employ their inherent chemical reactivity to interact with residual gases through stoichiometric reactions, thereby promoting effective outgassing [54]. According to the reported literature, representative chemical getters include CuO, AgO, and CaO [55,56,57,58]. The third classification involves physicochemical getters that combine physical adsorption and chemical absorption mechanisms. These materials initially sequester gas molecules through physisorption at the surface, subsequently causing molecular dissociation into atomic species. Then, atomic diffusion into the bulk matrix occurs through a temperature-dependent process governed by the crystallographic structure and Fickian diffusion parameters of the getters [30]. The zirconium–vanadium––iron (Zr-V-Fe) alloys and barium–lithium (Ba-Li) intermetallic compounds are extensively studied due to their synergistic adsorption–absorption capabilities [30,59,60,61].

## 3. Design Strategy for Getters in VIPs

The high-vacuum environment within VIPs is necessary for maintaining the thermal insulation performance. An ideal getter material for VIPs must balance a high sorption performance with physical, chemical and economic factors to guarantee the efficiency, durability and cost effectiveness of the VIP. Given these issues, the creation of innovative materials with suitable structures is an industrial challenge to enhance absorption capacity of the getter. The following approaches can be considered.

### 3.1. Nanostructured Materials

Porous nanostructured materials offer a remarkable specific surface area, which facilitates abundant adsorption sites to significantly enhance gas adsorption capacity, improving the gas absorption efficiency of the getter. Typical materials, such as activated carbon [35,36] and zeolites [27,29,53], achieve specific surface areas ranging from 1000 to 10,000 m^2^/g, rendering them widely employed as getters.

**Figure 2:**
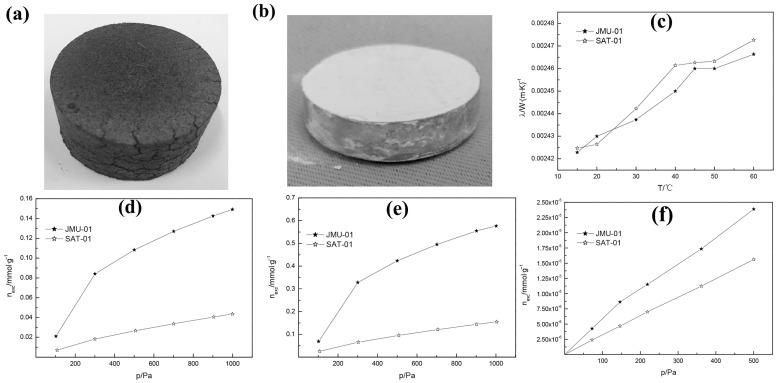
Photos of JMU-01 (**a**) and SAT-01 (**b**). (**c**) Temperature dependence of thermal conductivities of VIPs composed of JMU-01 and SAT-01. Isotherms of ethylene (**d**), propylene (**e**) and hydrogen (**f**) adsorptions at 273.15 K. This figure was reproduced with permission from ref. [35].

Carbon materials, recognized as a class of porous materials widely utilized in various applications, are characterized by an extensively developed porous architecture and an exceptionally high specific surface area. Yu et al. [36] used activated carbon as a getter to offer strong adsorption for H_2_O, proving to be a promising storage medium for alkenes, hydrocarbons and H_2_. Zheng et al. [35] developed a carbon-based getter for glass fiber core VIPs. The activated carbon sample is subjected to the surface modification using transitional metal doping, which was then mixed with an equivalent mass of expanded graphite (ENG) to form the getter (JMU-01) (Figure 2a). A control sample was prepared by combining the commercial desiccant with lithium–barium alloy composite getter (SAT-01) (Figure 2b). Both samples exhibit a comparable initial thermal conductivity (~2.45 mW/(m·K) at 15 °C) and nearly identical temperature-dependent behavior within the operational range of 15–60 °C (Figure 2c). However, compared to SAT-01, the hierarchically porous carbon-based composite demonstrates a superior hydrocarbon adsorption capacity, achieving maximum adsorption increments of 242.42%, 272.65% and 85.24% for ethylene, propylene and hydrogen, respectively (Figure 2d–f). Efficient gas scavenging is critical to mitigate vacuum degradation caused by outgassing. Carbon-based getters with improved selectivity for hydrocarbon positions are regarded as promising candidates for next-generation bio-based core materials, aligning with sustainability objectives [62,63,64,65].

**Figure 3:**
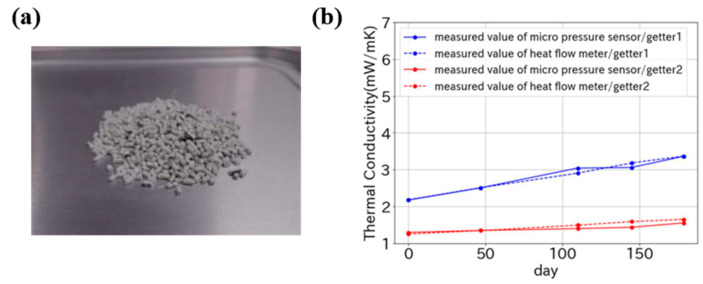
(**a**) Photo of zeolite getter. (**b**) Comparative long-term performance of Getter1 and Getter2. This figure was reproduced with permission from ref. [53].

Nanostructured materials offer notable advantages including tunable pore architecture and molecular selectivity. The getters designed demonstrate selective adsorption capabilities for target gas species through surface functionalization and the precise modulation of pore dimensions (micropores: <2 nm, mesopores: 2–50 nm, macropores: >50 nm). For instance, zeolite molecular sieves have precisely engineered pore architectures (0.3–1.0 nm), enabling molecular discrimination through the size-selective adsorption of small gas molecules [66]. In a comparative analysis by Yamamoto et al. [29,53], two zeolite-based getters (designated as Getter1 and Getter2) were evaluated for their adsorption performance in VIPs. The photo of a zeolite getter in Figure 3a demonstrates the granular variant employing binder-assisted granulation to improve processability. The absorption efficiency of Getter2 under low-pressure conditions is higher than that of Getter1, indicating an enhanced gas adsorption capacity, mainly due to structural variations in its framework topology. Notably, Getter2 exhibits a lower initial thermal conductivity (1.3 mW/(m·K) than 2.2 mW/(m·K) of Getter1 at 20 °C) and enhanced long-term reliability (Figure 3b). Araki et al. [27] performed comparative validation studies on the conventional molecular sieve 13X (MS13X) and a hydrophobic synthetic zeolite (HiSiv™). The HiSiv™-based getter showed a superior adsorption efficiency under accelerated aging at 105 °C compared to its MS13X counterpart. This differential performance stems primarily from HiSiv™’s engineered features of hydrophobic surface modification and hierarchical pore architecture, which mitigate competitive water vapor adsorption. Zeolite-based getters with precisely engineered characteristics (e.g., controlled hydrophobicity, optimized pore geometry, and surface functionalization) can be systematically developed to align with transient gas emission patterns in VIP systems. Such material optimization pathways not only enhance thermal performance but also extend service lifetimes by 30–50%, addressing a key bottleneck in next-generation VIP applications ranging from building insulation to low-temperature supply chain applications [1,67].

**Figure 4:**
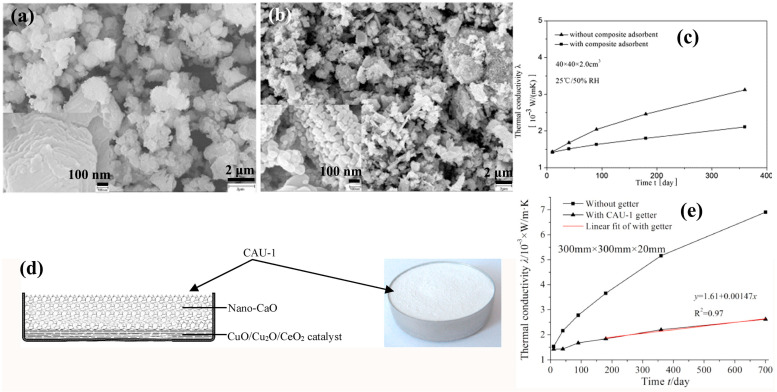
SEM images of the modified CaO (**a**) and modified CuO (**b**). (**c**) The test results of long-term performance with and without composite getter of nano-active CaO/CuO. This figure was reproduced with permission from ref. [12]. (**d**) Schematic diagram and photograph of the composite getter of CAU-1. (**e**) Tracking test results of VIP thermal conductivity over time with and without a getter. This figure was reproduced with permission from ref. [14].

Nanostructure engineering enables gas adsorption in porous materials, where the adsorption properties of these materials are mainly described by the Langmuir model of monolayer adsorption [34,68,69]. Under this model, adsorption occurs primarily physical interaction between gas molecules and adsorbent surfaces. However, such physisorption mechanisms are insufficient for essential atmospheric components (N_2_ and O_2_), which fail to fulfill the criteria of the optimal adsorption state. The systematic development of enhanced chemisorption capabilities consequently emerges as a critical research frontier. Di et al. [12] synthesized a composite getter comprising nano-active CaO and CuO, as shown in Figure 4a,b. The CaO component rapidly adsorbs residual H_2_O and CO_2_ in VIPs, while CuO catalytically oxidizes H_2_ and CO into H_2_O and CO_2_, which CaO subsequently sequesters. The CaO component actively scavenges H_2_O through hydration (CaO + H_2_O → Ca(OH)_2_) and CO_2_ via carbonation (CaO + CO_2_ → CaCO_3_), while the CuO/Cu_2_O redox pair synergistically catalyze H_2_ oxidation (2H_2_ + O_2_ → 2H_2_O), effectively mitigating hydrogen-induced VIP degradation [12,70]. When integrated into glass fiber-core VIPs, the composite getter exhibits a lower initial thermal conductivity than conventional systems, achieving a superior long-term performance (Figure 4c). Their service life has been dramatically improved to 10–15 years, which can support their application in refrigeration and cryogenics. Similarly, transition metal oxides, such as cerium oxide (CeO_2_) and cobalt oxide (Co_3_O_4_), have emerged as attractive getter materials, which have gained increasing attention due to their abundant availability, tunable surface chemistry and unique catalytic activity towards gas adsorption [14,45,71]. In particular, the variable oxidation states and surface defect structures of transition metal oxides enable the effective chemisorption of residual gases (e.g., O_2_, N_2_, and H_2_O) through redox reactions, which is crucial for maintaining long-term vacuum stability in thermal insulation applications. Based on their previous results, Di et al. [14] introduced a novel composite getter comprising CaO and CeO_2_ (CAU-1) (Figure 4d), in which the VIP samples were fabricated using hybrid encapsulations and a core composed of chopped fiberglass strands. After a period of 2 years, the VIPs containing the CAU-1 getter exhibited an increased thermal conductivity of 1.2 mW/(m·K) (Figure 4e). Accelerated aging simulations projected an increase in the VIP center-of-panel thermal conductivity to 15 mW/(m·K) after 25 years. In short, they pioneered a dual-function getter system combining chemisorption (CaO) and catalytic conversion (Cu/Ce/Co oxides) to offer a viable pathway for building-integrated VIPs requiring >20-year service life.

### 3.2. Advanced Getter Materials

**Figure 5:**
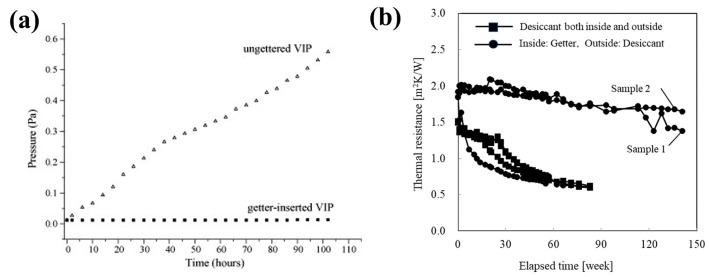
(**a**) Pressure increases in the VIPs with and without getters. This figure was reproduced with permission from ref. [11]. (**b**) Change in thermal resistance in the accelerated test using the double envelope type getter. This figure was reproduced with permission from ref. [17].

It is important to explore alloys or composites with a greater affinity for residual gases, contributing to improving long-term vacuum stability and mitigate performance decay. Conventional zirconium (Zr) or barium (Ba) getters have been widely used in the vacuum field [72,73]. However, their application in VIPs is subject to a poor gas sorption capacity and high activation temperature thresholds [74] since the thermal stability of VIP polymer laminates cannot be maintained above 150 °C [75]. In contrast to traditional getters, Ba-Li alloys facilitate the adsorption of active gases under ambient setting, bypassing the energy-intensive thermal activation process [37]. Consequently, a multi-component COMBOGETTER getter consisting of CaO, BaLi_4_ and Co_3_O_4_ has been developed by the SAES Getters Company [37]. Also, Nanjing Shangong New Material Technology Co., Ltd. (Thanko) has introduced the TK107 series, a novel formulation incorporating CaO, BaLi_4_ and ZrVFe [76]. These advanced getters demonstrate efficacy in adsorbing ambient H_2_O and reactive gases under operational conditions. Kwon et al. [11] highlighted the COMBOGETTER getter as a commercially available getter material, referencing manufacturer-reported performance metrics that include a total sorption capacity exceeding 1.33 × 10^3^ Pa·L and an initial sorption rate greater than 1.33 × 10^−4^ Pa·L/s. Figure 5a demonstrates the comparative pressure evolution profiles of VIPs with and without integrated getter systems. The presence of getter materials helps to maintain a stable internal pressure, contrasting with the rapid pressure rise in panels without integrated getter. Thus, these results indicate that VIP systems incorporating this getter can achieve operational lifetimes surpassing 20 years. Similarly, Katsura et al. [17] demonstrated promising results by integrating SAES getter materials with a double-envelope VIP configuration. Accelerated aging tests were conducted by exposing VIP specimens to 80 °C environments, a prototype featuring interior getter placement with external desiccant configuration exhibited merely 10.5% thermal resistance degradation after 141 weeks (Figure 5b). The application of getter materials and dual barrier envelopes significantly mitigates pressure differentials and gas permeation within the inner VIP, thereby extending service lifetimes. However, these commercial alloy-containing getters remain sparingly employed in VIP applications owing to the risk of combustion [59,77]. When exposed to ambient moisture, these alloys undergo exothermic hydrolysis reactions that pose combustion risks. Furthermore, their hydrogen absorption mechanism forms metastable solid solutions instead of stable hydrides, which leads to reversible storage conditions for absorbed hydrogen under thermal stress [78]. H_2_ significantly impairs the insulation performance of VIPs because its thermal conductivity is 7 times that of air [40,79]. Therefore, alloy-based compounds are generally not utilized independently as getters but rather in conjunction with desiccants and hydrogen-absorbing substances.

### 3.3. Electrochemical Getters

To address the limitations of conventional getter materials in VIPs, particularly their restricted gas sorption capacity and safety concerns, electrochemical getters (EGs) have emerged as an innovative high-efficiency solution. Jebaraj et al. [38] pioneered a novel electrochemical device architecture comprising a lithium foil or lithiated metal oxide anode, a polyethylene oxide (PEO)-based polymer electrolyte incorporated with lithium salts and a porous nickel cathode, which are organized in a multilayered configuration. Experimental observations reveal distinct electrochemical behaviors of lithium-based getters across varied atmospheres. Ar does not react with metallic lithium, exhibiting the lowest open-circuit potential (0.75 V). Residual gases in high-vacuum (HV) conditions induce measurable reactions, yielding a slightly elevated potential, compared to Ar. The explanation of this rational is that the N_2_ and O_2_ gases exhibit a substantial reactivity, generating significantly higher potential outputs. The morphological evolution of lithium metal on the electrode surface under an Ar atmosphere, accompanied by evident product formation, provides conclusive evidence that electrochemically generated metallic lithium actively interacts with all atmospheric molecular species through gettering processes. Notably, such gas-absorbing behavior holds significant implications for VIPs. It is critical for maintaining ultralow internal pressure and eliminating residual gases to achieve optimal thermal insulation performance. Additionally, this breakthrough structural design facilitates three essential advancements, such as the incorporation of an inherently safe on-demand lithium activation mechanism, high-capacity polymer architecture and intelligent gas leakage monitoring capabilities. These innovations fundamentally alter traditional getter applications in VIP systems by sustaining ultrahigh vacuum levels (10^−1^–10^−2^ Pa) over extended periods. Therefore, EGs have emerged as very attractive candidates for next-generation gas absorption materials, demonstrating an exceptional potential to transform gas management in vacuum insulation systems.

## 4. Challenges and Perspectives

**Figure 6:**
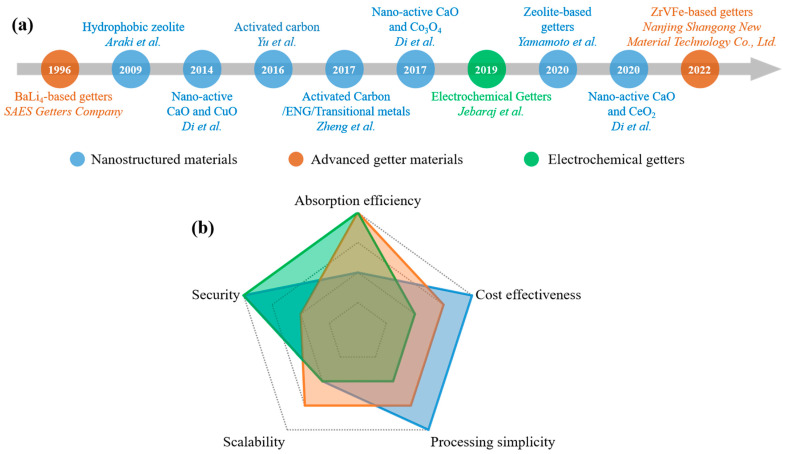
Summary and comparison of various treatment strategies for getter materials. (**a**) Roadmap of major achievements in the field of getter for VIPs. (**b**) An evaluation of various methods from five practical application metrics: absorption efficiency, cost effectiveness, processing simplicity, scalability, and security [12,14,27,29,35,36,38,45].

In summary, this review comprehensively analyzes the strategic deployment of advanced getter materials in VIPs to elucidate their critical function in preserving vacuum integrity (<5 Pa operational pressure), ensuring long-term thermal performances. The application of getter materials in vacuum insulation panels (VIPs) traces its origins to pioneering work by SAES Getters in 1996, who first implemented metallic getters for gas adsorption in evacuated panels [37]. However, early adoption faced significant commercialization barriers due to prohibitive manufacturing costs and safety concerns associated with pyrophoric alloy components. Subsequent advancements in core material optimization (e.g., glass fiber) and barrier film engineering (multilayer metallized polymer laminates) enabled cost reductions of 40–60% through scaled production, facilitating initial deployment in premium refrigeration. This technological inflection catalyzed intensive research into next-generation getter systems. Current development strategies, systematically categorized in Figure 6a, emphasize three principal approaches: nanostructured materials, advanced getter materials and electrochemical getters. A comparative evaluation was carried out across five critical operational parameters—absorption efficiency, cost effectiveness, processing simplicity, scalability, and security (Figure 6b). Nanostructured adsorbents exhibit superior cost effectiveness and inherent safety (non-pyrophoric), though they are constrained by moderate gas uptake capacities. Advanced alloy systems (e.g., BaLi_4_/ZrVFe composites) demonstrate exceptional multi-gas absorption through hybrid chemisorption mechanisms, yet require encapsulation technologies to mitigate hydrolysis risks. Electrochemical getters present unique advantages in terms of absorption rate regulation and real-time vacuum monitoring via electric signals, though their complex multilayer architectures (Ni/PEO/Li foil) incur higher cost premiums versus conventional systems. Strategic material selection must consider application-specific requirements: cost-sensitive construction insulation favors hierarchical carbon-based getters, while performance-critical medical applications necessitate electrochemical getters.

State-of-the-art getter systems, particularly nanostructured materials and advanced alloys materials and surface-modified composites, leverage their tunable chemical affinity and bimodal pore architectures to achieve synergistic gas capture through dual physisorption–chemisorption mechanisms. An ideal getter material for application in a VIP should possess the following properties: (1) atomic-level porosity engineering enabling selective adsorption of residual gases (H_2_O ≤ 10 ppm, CO_2_ ≤ 100 ppm); (2) an exceptionally high specific surface area (>1000 m^2^/g) to maximize adsorption sites and gas molecule interaction; (3) surface functionalization with transition metal catalysts enhancing dissociation kinetics of reactive gas species; and (4) an environmentally sustainable composition with non-toxic degradation products, ensuring full compliance with RoHS directives and minimal ecological footprint throughout the lifecycle. These innovations have resulted in practical improvements in VIP performance. Field data from building envelope applications demonstrate service life extensions exceeding 20 years while maintaining thermal conductivity (λ-values) below 8 mW/(m·K), as evidenced by longitudinal studies on nanostructured materials used in getters in glass fiber-core panels [6,67]. Such a highly reliable VIP systems offer dual environmental benefits through significant carbon emission reduction and enhanced spatial efficiency.

However, several deficiencies and challenges must be resolved before actual application. (1) The development of high-precision in situ gas analysis systems is imperative to quantify gas speciation and partial pressure dynamics within VIPs, driving the rational design of intelligent getters with adaptive absorption profiles and facilitating the creation of gas-selective getters. (2) Metal–organic frameworks (MOFs) and covalent organic polymers (COPs) present promising potential as getters due to their exceptional specific surface areas (>7000 m^2^/g) and tunable pore architectures [80,81,82]. Catalytic properties can be engineered by metal cluster functionalization, facilitating synergistic gas capture-conversion mechanisms. Hierarchical pore architectures integrating micro-, meso-, and macropores exhibit a better improvement in gas diffusion kinetics compared to conventional zeolites. (3) Significant constraints remain in comprehending the surface reaction mechanisms of transition metal oxides in the presence of trace gas. State-of-the-art in situ transmission electron microscope (TEM) studies reveal that oxygen vacancy migration in Co_3_O_4_ nanostructures governs hydrogen oxidation efficiency, with surface reconstructions occurring at sub-nm scales [83]. A systematic investigation of dopant effects (e.g., Ce^3+^/Ce^4+^ ratios in ZrO_2_ catalysts) through combinatorial deposition techniques is required to optimize catalytic–adsorptive synergies [84,85]. (4) EGs exhibit great potential as next-generation sorption materials for VIPs, capable of achieving both the safe removal of internal gases and the real-time monitoring of internal pressure [86]. However, high production costs and immature technology currently hinder the industrial-scale commercialization of EGs. Getters are essential for guaranteeing the long-term service life and optimal energy efficiency of VIPs. Despite recent technological breakthroughs in getters, substantial challenges, such as cost effectiveness, safety profiles and adsorption efficiency, remain to be faced in complex gas environments. Therefore, future research requires interdisciplinary collaboration to integrate innovations in materials science with advanced engineering technologies, ultimately enabling the widespread application of VIPs in energy-efficient building systems, cold-chain logistics and various industrial fields.

## Figures and Tables

**Table 1:** Comparison of different gases composition in VIPs.

Core Material	Initial Gas Composition	Gas Composition After Aging (Without Getter)	Getter Material	Initial Thermal Conductivity mW/(m·K)	Thermal Conductivity After Aging mW/(m·K) @720 day	Ref.
Glass fiber	N_2_/O_2_/Ar/H_2_O	N_2_/O_2_/Ar/H_2_O/H_2_/CO/CO_2_/C_x_H_y_	CaO/CuO/Cu_2_O/CeO_2_	1.52	6.8	[14]
Glass fiber	N_2_/O_2_/H_2_/Ar/H_2_O/CO_2_	/	Zeolite	2.0	2.2	[34]
Glass fiber	Air/H_2_/ethylene and propylene	/	Activated Carbon /ENG/Transitional metals	2.45	/	[35]
Glass fiber	N_2_/O_2_/H_2_O/H_2_/Ar	H_2_/H_2_O/N_2_/O_2_/CO/CO_2_/Ar	Nano-CaO	2.2	5.8	[45]
			Nano-CaO/Co_3_O_4_	2.2	3.9	
			/	2.4	7.5	

## Data Availability

Data are contained within the article.
